# Understanding the unmet needs of patients with brain metastases referred for stereotactic radiotherapy and their caregivers: A prospective cohort study

**DOI:** 10.1093/nop/npaf105

**Published:** 2025-10-07

**Authors:** Fia Cialdella, Eva E van Grinsven, Steven H J Nagtegaal, Arthur T J van der Boog, Celeste C Hinkert, Marion G Jentink, Ernst J Smid, An Claes, Catharina E Kleynen, Tom J Snijders, Filip Y F de Vos, Marielle E P Philippens, Martine J E van Zandvoort, Szabolcs David, Joost J C Verhoeff

**Affiliations:** Department of Radiation Oncology, University Medical Center Utrecht, Utrecht (F.C., A.T.J.V.D.B., C.C.H., M.G.J., E.J.S., A.C., C.E.K., M.E.P.P., S.D.); Department of Medical Oncology, University Medical Center Utrecht, Utrecht (F.C., F.Y.F.d.V.); Department of Neurology & Neurosurgery, Brain Center, University Medical Center Utrecht, Utrecht (E.E.V.G., A.T.J.V.D.B., T.J.S., M.J.E.v.Z.); Department of Radiation Oncology, Erasmus Medical Center, Rotterdam (S.H.J.N.); Department of Radiation Oncology, University Medical Center Utrecht, Utrecht (F.C., A.T.J.V.D.B., C.C.H., M.G.J., E.J.S., A.C., C.E.K., M.E.P.P., S.D.); Department of Neurology & Neurosurgery, Brain Center, University Medical Center Utrecht, Utrecht (E.E.V.G., A.T.J.V.D.B., T.J.S., M.J.E.v.Z.); Department of Radiation Oncology, University Medical Center Utrecht, Utrecht (F.C., A.T.J.V.D.B., C.C.H., M.G.J., E.J.S., A.C., C.E.K., M.E.P.P., S.D.); Department of Radiation Oncology, University Medical Center Utrecht, Utrecht (F.C., A.T.J.V.D.B., C.C.H., M.G.J., E.J.S., A.C., C.E.K., M.E.P.P., S.D.); Department of Radiation Oncology, University Medical Center Utrecht, Utrecht (F.C., A.T.J.V.D.B., C.C.H., M.G.J., E.J.S., A.C., C.E.K., M.E.P.P., S.D.); Department of Radiation Oncology, University Medical Center Utrecht, Utrecht (F.C., A.T.J.V.D.B., C.C.H., M.G.J., E.J.S., A.C., C.E.K., M.E.P.P., S.D.); Department of Radiation Oncology, University Medical Center Utrecht, Utrecht (F.C., A.T.J.V.D.B., C.C.H., M.G.J., E.J.S., A.C., C.E.K., M.E.P.P., S.D.); Department of Neurology & Neurosurgery, Brain Center, University Medical Center Utrecht, Utrecht (E.E.V.G., A.T.J.V.D.B., T.J.S., M.J.E.v.Z.); Department of Medical Oncology, University Medical Center Utrecht, Utrecht (F.C., F.Y.F.d.V.); Department of Radiation Oncology, University Medical Center Utrecht, Utrecht (F.C., A.T.J.V.D.B., C.C.H., M.G.J., E.J.S., A.C., C.E.K., M.E.P.P., S.D.); Department of Neurology & Neurosurgery, Brain Center, University Medical Center Utrecht, Utrecht (E.E.V.G., A.T.J.V.D.B., T.J.S., M.J.E.v.Z.); Department of Experimental Psychology and Helmholtz Institute, Utrecht University, Utrecht (M.J.E.v.Z.); Department of Radiation Oncology, University Medical Center Utrecht, Utrecht (F.C., A.T.J.V.D.B., C.C.H., M.G.J., E.J.S., A.C., C.E.K., M.E.P.P., S.D.); Department of Radiation Oncology, Cancer Center Amsterdam, Amsterdam University Medical Center, Amsterdam (S.D., J.J.C.V.); Department of Radiation Oncology, Cancer Center Amsterdam, Amsterdam University Medical Center, Amsterdam (S.D., J.J.C.V.)

**Keywords:** brain metastases, radiotherapy, quality of life, caregivers, cohort studies

## Abstract

**Abstract:**

BackgroundBrain metastases (BMs) affect >30% of patients with cancer. Beyond survival, quality of life (QoL), and disease progression are critical concerns for both patients and their caregivers. We present the design of COIMBRA (Cohort for patient-reported Outcomes, Imaging, and trial inclusion in Metastatic BRAin disease), clinical characteristics, and pre-radiotherapy QoL data for patients and caregivers.

**Methods:**

COIMBRA is an observational, prospective, single-center cohort at UMC Utrecht, the Netherlands, focusing on BMs patients planned for (fractionated) stereotactic radiosurgery (SRS) and their caregivers. We collected clinical characteristics, imaging, and QoL (patient and caregiver) data via patient-reported outcome (PRO) questionnaires. PROs include the EORTC QLQ-C30, BN20, EQ-5D-3L (including VAS), CFQ, HADS, MFI, and CSI. PROs were compared with reference values from the literature for healthy, cancer, and brain tumor populations.

**Results:**

From April 2019 to April 2023, 377 patients (50.4% male, median age 66 years) who received stereotactic radiosurgery, were included. Two hundred and seventy-six patients and 115 caregivers consented to PROs. Patients reported lower QoL compared to healthy individuals and primary brain tumor patients, with worse role- and cognitive functioning compared to patients with cancer. Pre-radiotherapy, 1/3 of the caregivers felt overwhelmed. Almost 40% of them report signs of anxiety, which is less than caregivers of cancer or primary brain tumor patients.

**Conclusions:**

These data from a large cohort of patients with BMs and their caregivers underscore the unique clinical profile of this vulnerable population. These findings highlight the urgent need for targeted support systems to address unmet needs of both patients and caregivers.

Key points

- COIMBRA observational trial investigates QoL of BMs patients and their caregivers.
- Patients with BMs have low QoL, similar to those with primary brain or other cancers.
- One-third of caregivers are already overwhelmed pre-radiotherapy and 40% report anxiety.

Importance of the study Brain metastases (BMs) now affect over 30% of cancer patients, and with advances in primary tumor treatment and imaging their incidence continues to rise, yet their impact on quality of life (QoL) and caregiver well-being remains under-recognized in practice. This study provides a comprehensive overview of the QoL and clinical characteristics of patients with BMs and their caregivers prior to stereotactic radiosurgery by collecting patient-reported outcomes (PROs) and clinical data including imaging and cognitive functioning. Patients experienced impairments in multiple areas, including physical, emotional, and cognitive functioning, with high levels of fatigue. Caregivers, despite good overall health status, showed signs of anxiety and experienced high burden. These findings enable personalized treatment planning, targeted symptom management, and early psychosocial support, directly informing clinical workflows. Moreover, this cohort supports an efficient infrastructure for future randomized trials according to the of Trials within Cohorts (TwiCs) design, potentially accelerating advancements for BMs patients.

Brain metastases (BMs) present a significant challenge in oncology, affecting both the quality of life (QoL) and survival outcomes of cancer patients. Most BMs originate from lung cancer, breast cancer, or melanoma and approximately 2-14% of the cases have an unknown primary tumor.^1^^,^^2^ BMs occur in 10-40% of all patients with cancer, with incidence expected to increase due to improved diagnosis and longer survival due to systemic therapies.^3^ Median survival varies depending on several factors: primary tumor, Karnofsky Performance Status (KPS), number of BMs, and the presence of extracranial metastases. Untreated, survival rates range from 2 to 3 months, extending to 3 to 18 months with treatment.^2^^,^^4^^,^^5^

Treatment options for BMs include surgery, systemic therapy, radiotherapy, or a combination. Non-prophylactic radiotherapy options are stereotactic radiosurgery (SRS) and whole-brain radiation therapy (WBRT). SRS delivers precise, localized radiation in 1-3 fractions (15-24 Gy), minimizing exposure to healthy tissue.^3^ WBRT uses a lower biological dose (e.g. 5 × 4 Gy or 10 × 3 Gy), delivered to the entire brain.^6^^,^^7^ In the Netherlands, SRS is preferred for patients with ≤10 BMs and a total volume ≤30 cm³, WBRT is considered for patients with >10 metastases or larger volumes.^3^ Prophylactic cranial irradiation (PCI) may be used for small cell lung cancer to prevent BMs (20-25 Gy in 2.5-4 Gy fractions).^8^ Whenever possible, SRS is preferred over WBRT to spare healthy brain to minimize side effects and preserve QoL, particularly cognitive function. However, caution is advised with radiation for patients with a KPS below 70. Healthcare providers may prioritize QoL over lifespan in vulnerable patients, and opt for best supportive care (BSC).^3^

Improving QoL of patients with BMs requires new treatment and rehabilitation options. These include access to palliative care for symptom relief, psychological support, and rehabilitation services to address cognitive deficits, motor deficits, and fatigue.^9^^,^^10^ However, the understanding of these patients’ unmet needs is limited. Better knowledge can guide the development of comprehensive, multidisciplinary models of care that address the diverse needs of patients with BMs, ultimately improving both survival and QoL.^11^^,^^12^ Effective patient-centered care requires better communication and coordination among healthcare teams, especially when cognitive impairment affects patients’ ability to participate in care decisions.^13^ Additionally, we need more knowledge about the QoL before radiotherapy and the clinical characteristics of patients with BMs. Historically, patients with BMs have often been excluded from clinical trials. Recent recommendations from the ASCO-Friends of Cancer Research Brain Metastases Working Group advocate for modernizing eligibility criteria to better represent patients with BMs in clinical research. Despite this, few studies have investigated this topic comprehensively, often using small samples or focusing solely on primary brain tumors.^13^^,^^14^ Caregivers of patients with BMs also face unique challenges, related to both BMs and the primary tumors, profoundly affecting caregivers QoL.^15^^,^^16^ Addressing these challenges requires new perspectives and updated descriptions of the patient population to improve our understanding of clinical needs and QoL outcomes. This includes larger surveys and expanding research beyond standard questionnaires (such as C30, BN20, EQ-5D), targeting critical unmet needs in this patient group, such as cognitive impairment, fatigue, and caregiver burden.

Pre-radiotherapy QoL is heavily influenced by disease characteristics and the patients’ overall health status.^14^ The symptom profiles vary considerably among patients. The intricate relationships between lesion, brain structure, and dysfunction are essential to understand the various clinical presentations. To accurately map lesion-symptom relationships in patients with BMs, high-quality, multi-sequence MRI scans (T1-weighted with and without contrast enhancement, and T2-FLAIR) are required. These imaging modalities enable precise delineation of various abnormalities including peritumoral edema, necrotic core, and tumor core. Advanced image analysis tools, such as voxel wise and dysconnectivity analyses, are subsequently needed to process and interpret the acquired MRI data.^17^

The gold standard for clinical trials is the randomized controlled trial (RCT), yet these trials often face challenges such as target inclusion failures, highly selective study populations, and limited generalizability. To address these issues, the concept of Trials within Cohorts (TwiCs) was developed, offering a more efficient and patient-centered approach.^18^^,^^19^ This method, previously known as cohort multiple Randomized Controlled Trial (cmRCT), involves creating an observational prospective cohort of patients undergoing standard treatment for a specific condition.

The “Cohort for patient-reported Outcomes, Imaging and trial inclusion in Metastatic BRAin disease” (COIMBRA) is a prospective observational cohort that collects data from patients with BMs and their caregivers, assessing QoL, unmet needs, and caregiver burden. Unlike prior studies focused solely on patients or limited outcome measures, this cohort combines clinical data, imaging, and patient- and caregiver-reported outcomes within a TwiCs framework.^20^ This design contrasts with standard observational cohorts by enabling future pragmatic RCTs within the same real-world population. At inclusion, participants provide advance consent for potential randomization, eliminating repeated re-consenting. Patients randomized to the intervention arm may receive new treatments, while control patients continue standard care without additional burden or trial notification. Routinely collected cohort data serve as control outcomes, creating an efficient, flexible infrastructure for embedding multiple RCTs and imaging sub-studies without additional recruitment barriers.

The aim of this work is to provide a detailed description of the COIMBRA design as well as to present clinical characteristics and QoL assessments for a large sample of patients with BMs and their caregivers before SRS.

## Methods

COIMBRA forms a broad observational framework including patients receiving various types of cranial radiotherapy. This article focuses on a COIMBRA sub-study including only those treated with fractionated SRS, excluding patients who underwent WBRT or PCI, and specifically describes the study sample, baseline symptom burden, and QoL in patients and caregivers, and explores factors influencing QoL and global functioning. Below, we first describe the overall COIMBRA design, followed by specific methods for this sub-study.

### Study Design of the COIMBRA Cohort

The COIMBRA cohort, designed using TwiCs principles, has 2 main objectives: first, to collect prospective clinical data and patient-reported outcomes (PROs); second, to establish an efficient infrastructure for RCTs and imaging studies. Patients referred for radiotherapy at UMC Utrecht were invited to participate, if they had histologically confirmed or radiologically suspected BMs, or an indication for PCI. Exclusion criteria were: age < 18 years, inability to consent due to mental or cognitive impairment, severe psychiatric conditions, or insufficient Dutch language proficiency. The study protocol was approved by the local Institutional Review and Ethics Board (approval number 18-642, ClinicalTrials.gov identifier NCT05267158).^20^

**Figure 1.**
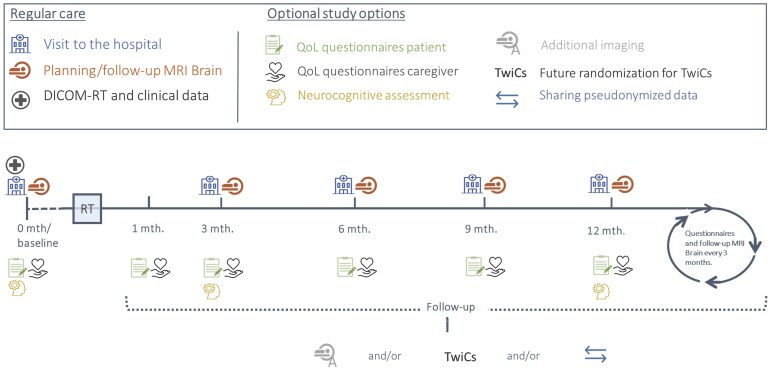
Timeline of the patient enrollment and data collection in COIMBRA. COIMBRA study timeline depicting patient enrollment, data collection, and follow-up activities. The figure shows key patient visits, MRI scans, and optional assessments including quality of life (QoL) questionnaires and neurocognitive tests. Pre-radiotherapy (baseline) data collection includes patient demographics, oncological history, brain metastases characteristics, and treatment information. Follow-up activities may include additional MRI scans, randomization within the TwiCs framework, and/or pseudonymized data sharing, subject to participant consent obtained at baseline. RT = radiotherapy.

Inclusion started on April 2019. After radiotherapy intake, researchers presented the study and obtained written consent. All patients could consent to clinical data, and optionally to 5 subparts of COIMBRA: (1) filling in QoL questionnaires, (2) performing a neurocognitive assessment (NCA), (3) contributing in additional image data acquisition, (4) participating in future randomization for TwiCs, and (5) sharing their pseudonymized data with third parties. Details of clinical data and QoL data usage are explained in the following sections. The other subparts are described in Supplementary Material Section Text S1. Figure 1 shows the timeline of enrollment and data collection in COIMBRA.

#### Clinical Data

Researchers prospectively collected observational data including demographics, oncological history, BMs characteristics, treatment data, and clinical outcomes and symptom data, extracted from radiotherapy intake notes. Survival data were calculated from the first radiotherapy date. If patients received radiotherapy before enrollment, the earlier date was used. Data were gathered from electronic patient records (HiX, Chipsoft, The Netherlands) and referral letters.

Brain MRI scans were acquired as part of routine clinical care for all patients, conducted both before and after radiotherapy. At the UMCU, follow-up scans are usually performed every 3 months post-radiotherapy, with additional scans administered if patients exhibited symptoms earlier. The MRI protocol included T1-weighted imaging both with and without gadolinium contrast, and T2-FLAIR. Concurrently, planning CT was used to calculate the radiation dose distribution and the treatment plans. For the current study, we analyzed the high-prevalence locations (also called: “hotspots” of lesions) using the FSL tool “autoaq” and 3 brain atlases.^21–23^  Supplementary Material Section Text S2 contains the detailed information on the MR image analyses methodology.

#### QoL of Patients and Their Caregivers

In order to obtain more detailed information, patients could additionally consent to fill in PROs questionnaires. These were sent to the patient before the start of radiotherapy (baseline), after 1 month, after 3 months, and then every 3 months. Starting June 2021, patients could also consent to have similar questionnaires sent to their caregivers on the same schedule. Participants could choose between paper questionnaires or electronic submission via PROFILES (Patient-Reported Outcomes Following Initial treatment and Long-term Evaluation of Survivorship).^24^

Validated questionnaires were selected to capture outcomes relevant to BMs including general health-related quality of life (EORTC QLQ-C30), brain cancer-specific symptoms (EORTC QLQ-BN20), cognitive function (CFQ), health status (EQ-5D-3L), anxiety and depression (HADS), and fatigue (MFI).^25–31^ Additionally, 2 questionnaires were used to assess personality traits (NEO-FFI) and coping styles (UCL) at baseline. For caregivers, questionnaires were chosen to assess aspects relevant to their QoL and burden, including caregiver strain (CSI), symptoms of anxiety and depression (HADS), and general health status (EQ-5D-3L).^27^^,^^30^^,^^32^^,^^33^ Similar to patients, the NEO-FFI and UCL were used only once pre-radiotherapy to collect data on caregivers’ personality and coping style.^34^^,^^35^ Detailed information on each questionnaire is provided in Supplementary Table S1.

The results of the PROs were compared to reference values drawn from published studies including, healthy individuals, BMs, cancer, and/or primary brain tumor populations.^9^^,^^26^^,^^27^^,^^36–58^ For each questionnaire and outcome domain, we selected the most relevant reference populations based on sample composition, sample size, and availability of detailed statistical data (means, standard deviations, and clinically validated cutoff points). This systematic selection enhances the clinical relevance and interpretability of comparisons, while acknowledging differences in exact disease profiles. Our approach aligns with recommended best practices emphasizing the representativeness and contextual validity of normative data.^59^ All PRO scores, from our cohort and reference studies, were rounded to one decimal place for consistency throughout the article.

### Data Collection and Statistics of the Pre-Radiotherapy Characteristics

#### Data Collection

For this sub-study within COIMBRA, we analyzed data collected from April 2019 to April 2023, focusing only on patients who received (fractionated) SRS. Baseline characteristics were prospectively collected, as detailed in Section “Clinical Data”. Continuous variables were reported as median (IQR), while PROs were reported as mean (SD) scores, except for the CFQ, which is reported as both median (IQR) and mean (SD) to enable the comparison with literature values. NEO-FFI and UCL questionnaires were excluded from this analysis due to their substantial data volume, and these tests focus on personality and coping rather than QoL.

#### Statistical Analysis

##### Statistical Methods

The Pearson’s correlation coefficient was used to evaluate the strength and direction of the relationships between total scores and domain scores from the questionnaires. The interpretation of the correlation coefficients followed the guidelines provided by Chan.^60^ The correlation coefficients were categorized as follows: no correlation *r* < 0.3, fair correlation: 0.3 ≤ *r* < 0.50, moderately strong correlation: 0.6 ≤ *r* < 0.80, and very strong correlation: *r* ≥ 0.8. Additionally, false discovery rate (FDR) correction was applied using the Benjamini-Hochberg method to adjust for multiple comparisons, ensuring the control of false positives.^61^ All analyses were conducted using IBM SPSS for Windows, version 25.

## Results

### Inclusion and Consent

**Figure 2.**
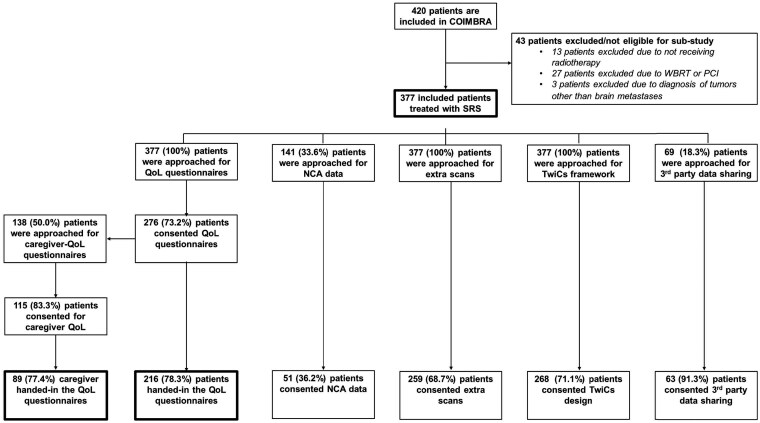
Flowchart of patient consent in different parts of the COIMBRA study. This flowchart illustrates the process of obtaining patient consent for various components of the COIMBRA project. Boxes with thick borders indicate patients included in this sub-study. Numbers indicate absolute counts; percentages represent proportions relative to the relevant parent group or total cohort specified. Abbreviations: NCA = neurocognitive assessment; PCI = prophylactic cranial irradiation; SRS = stereotactic radiosurgery; TwiCs = trials within cohorts; WBRT = whole-brain radiotherapy; QoL = quality of life.

A total of 420 patients with BMs provided written informed consent to participate in COIMBRA from April 2019 to April 2023. Of these, 43 patients were excluded because they did not receive SRS, or had a diagnosis other than BMs. Among those included, 276/377 patients (73.2%) consented to fill out questionnaires. Caregiver questionnaires were introduced in June 2021, with 115 out of 138 approached patients (83.3%) consenting to this component. Additionally, 51 patients (36.2%) completed an NCA. Consent rates for additional MRIs and future randomization were 68.7% (259 patients) and 71.1% (268 patients), respectively. The patient inclusion flowchart is shown in Figure 2.

### Clinical Data

Among 377 included patients referred for SRS, 50.4% were male, median age of 66 years (IQR 58-72). The majority (51.7%) of the cohort presented with lung cancer as primary tumor. Extracranial metastases were present in 64.7%. Most patients had 1, 2, or 3 BMs (48.3%, 32.9%, and 14.1%, respectively). BMs were symptomatic in 69.2% of patients, and 8.5% had a KPS below 70. Chemotherapy and immunotherapy had been administered either previously or at the time of inclusion in 51.5% and 46.9% of patients, respectively, and targeted therapy in 15.6%. Brain surgery (including biopsy) before referral was performed in 27.6% of patients. Baseline patient and treatment characteristics are shown in Table 1.

**Table 1.** Baseline characteristics of the total cohort and subgroups by consent for patient-reported outcomes (PROs) and no consent for PROs.

		Total cohort	Consent for PROs	No consent for PROs
		N = 377	N = 276	N = 101
Sex, *n* (%)	Male	191 (50.4)	142 (51.4)	49 (48.5)
	Female	186 (49.6)	134 (48.6)	52 (51.5)
Age, years, median (IQR)		66 (58 - 72)	65 (57 - 72)	66 (59.5 - 73)
Previous brain radiotherapy, *n* (%)	Yes	46 (12.2)	34 (12.3)	12 (11.9)
	No	331 (87.8)	242 (87.7)	89 (88.1)
Primary tumor, *n* (%)	Lung	195 (51.7)	135 (48.9)	60 (59.4)
	NSCLC	181 (92.8)	126 (93.3)	55 (91.7)
	SCLC	14 (7.2)	9 (6.7)	5 (8.3)
	Breast	47 (12.5)	33 (12.0)	14 (13.9)
	Melanoma	44 (11.7)	35 (12.7)	9 (8.9)
	Gastro-intestinal	42 (11.1)	33 (12.0)	9 (8.9)
	Gynaecology	10 (2.7)	9 (3.3)	1 (1.0)
	Urological	29 (7.7)	21 (7.7)	8 (7.9)
	Other	5 (1.3)	5 (1.8)	0 (0.0)
	Unknown	5 (1.3)	5 (1.8)	0 (0.0)
Extracranial metastasis, *n* (%)	Yes	244 (64.7)	175 (63.4)	69 (68.3)
	No	133 (35.3)	101 (36.6)	32 (31.7)
Number of treated BMs, *n* (%)	1	182 (48.3)	134 (48.6)	48 (47.5)
	2	124 (32.9)	88 (31.9)	36 (35.6)
	3	53 (14.1)	43 (15.6)	10 (9.9)
	4-9	3 (0.8)	2 (0.7)	1 (1.0)
	10+	15 (4.1)	9 (3.7)	6 (6.0)
Symptomatic, *n* (%)	Yes	261 (69.2)	190 (68.8)	71 (70.3)
	No	116 (30.8)	86 (31.3)	30 (29.7)
KPS, *n* (%)	0-50	6 (1.6)	3 (1.1)	3 (3.0)
	60	26 (6.9)	12 (4.3)	14 (13.9)
	70	85 (22.5)	63 (22.8)	22 (21.8)
	80	121 (32.1)	91 (33.0)	30 (29.7)
	90	89 (23.6)	73 (26.4)	16 (15.8)
	100	34 (9.0)	25 (9.1)	9 (8.9)
	Missing	16 (4.3)	9 (3.3)	7 (7.0)
100%-PTV dose on largest brain metastasis, *n* (%)	1 × 24 Gy	64 (17)	47 (17.0)	17 (16.8)
	1 × 21 Gy	134 (35.5)	103 (37.3)	31 (30.7)
	1 × 18 Gy	108 (28.6)	80 (29.0)	28 (27.7)
	1 × 16 Gy	18 (4.8)	7 (2.5)	11 (10.9)
	1 × 15 Gy	47 (12.5)	37 (13.4)	10 (9.9)
	2 × 15 Gy	2 (0.5)	1 (0.4)	1 (1.0)
	3 × 8 Gy	4 (1.1)	1 (0.4)	3 (3.0)
Total median GTV volume, median (IQR) in mm^3^		7414 (2791 - 16 053)	7410 (2750 - 16 152)	7575 (2797 - 15 503)
Total median PTV volume, median (IQR) in mm^3^		10 301 (4354 - 20 631)	10 041 (4358 - 20 740)	10 998 (4317 - 19 773)
Any chemotherapy, *n* (%)	Yes	194 (51.5)	140 (50.7)	54 (53.5)
	No	183 (48.5)	136 (49.3)	47 (46.5)
Any immunotherapy, *n* (%)	Yes	177 (46.9)	126 (45.7)	51 (50.5)
	No	200 (53.1)	150 (54.3)	50 (49.5)
Any targeted therapy, *n* (%)	Yes	59 (15.6)	41 (14.9)	18 (17.8)
	No	318 (84.4)	234 (84.8)	83 (82.2)
Brain surgery, *n* (%)	Yes	104 (27.6)	80 (29.0)	24 (23.8)
	No	273 (72.4)	196 (71.0)	77 (76.2)

BMs = brain metastases; IQR = inter quartile range; KPS = Karnofsky performance status; NSCLC = non-small cell lung cancer; PROs = patient-reported outcomes; RTP = radiotherapy; QoL = quality of life; SCLC = small cell lung cancer.

**Figure 3.**
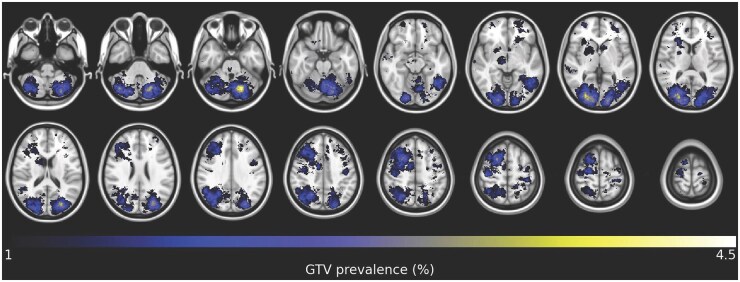
Spatial distribution of brain metastases (BMs) GTVs in stereotactic MNI space. The stereotactic overview illustrates specific brain areas with high-prevalence “hotspots” of lesions. The right side of the figure corresponds to the left side of the brain. The color bar ranges from cool (low prevalence) to warm color (high prevalence), according to the Gooch shading. To analyze the high-prevalence locations or “hotspots” of lesions, the FSL tool “autoaq” and three atlases were utilized.^21–23^

The spatially normalized distribution of BMs gross tumor volume (GTV) highlighted specific brain areas with relatively high-prevalence locations or “hotspots” of lesions. The highest prevalence was located in the cerebellum, notable in the left cerebellar lobe. In the supratentorial brain, hotspots were observed in both occipital lobes, the right precentral and postcentral gyri, the middle and superior frontal gyri, as well as the inferior temporal gyri. White matter fiber pathways with hotspots include the forceps major, middle cerebellar peduncle, vertical occipital fasciculus, optic radiations, superior longitudinal fasciculus, and frontal aslant tract on the right. Figure 3 shows the spatial distribution of BMs in our study population.

### QoL Outcomes

#### Patient QoL Prior Radiotherapy

Questionnaire return rate among patients who consented to the collection of PROs was 78.3% at baseline. Table 2 shows the detailed QoL outcomes of COIMBRA patients compared to reference populations.

**Table 2.** COIMBRA—patient-reported outcomes (PROs) compared to reference scores of healthy, cancer, and brain tumor populations.

Questionnaire	Domain/sub-score	COIMBRA patients	BMs population	Primary brain tumor population	Cancer population	Healthy population
EQ-5D^a^		N = 212		N = 1106	N = 534	N = 2367
	VAS, mean (SD)		n.a.	68.2	68.0	82.0
C30^b^		N = 198	N = not reported	N = not reported	N = not reported	N = 1000
	Global health status, mean (SD)	64.9 (20.0)	59.4	61.9	61.3	77.4
	Physical functioning, mean (SD)	74.3 (20.3)	74.9	79.2	76.7	90.7
	Role functioning, mean (SD)	59.9 (33.3)	75.0	67.4	70.5	89.1
	Emotional functioning, mean (SD)	74.9 (19.4)	71.9	70.4	71.4	82.3
	Cognitive functioning, mean (SD)	77.5 (22.7)	82.0	71.5	82.6	90.3
	Social functioning, mean (SD)	73.6 (25.9)	68.5 (N = 9)	73.1	75.0	91.9
	Financial problems, mean (SD)	5.7 (15.8)	29.6 (N = 9)	16.9	16.3	4.9
HADS^c^		N = 208		N = 190	N = 2625	N = 315
	Anxiety score, mean (SD)	4.7 (3.6)	n.a.	7.6	4.6	3.5
	Depression score, mean (SD)	4.1 (3.6)	n.a.	7.2	4.4	3.8
	Signs of anxiety, %	20.2	n.a.	36.3	20.9	11.8
	Signs of depression, %	19.2	n.a.	32.6	19.0	12.8
MFI^d^		N = 202		N = 31	N = 1818	N = 2512
	Total score, mean (SD)	57.9 (17.8)	n.a.	n.a.	54.8	44.9
	General fatigue score, mean (SD)	12.8 (4.5)	n.a.	13.4	12.2	9.8
	Physical fatigue score, mean (SD)	12.7 (4.8)	n.a.	11.6	12.2	8.8
	Reduced activity score, mean (SD)	12.4 (4.5)	n.a.	11.3	11.9	9.3
	Reduced motivation, mean (SD)	10.9 (4.0)	n.a.	9.5	8.9	8.7
	Mental fatigue score, mean (SD)	9.4 (3.8)	n.a.	12.8	9.6	8.3
BN20^e^		N = 215	N = 9	N = 745		
	Future uncertainty, mean (SD)	35.7 (22)	32.4	37.0	*n.a.*	*n.a.*
	Visual disorder, mean (SD)	14.8 (20.2)	21.0	12.8 (N = 746)	n.a.	n.a.
	Motor dysfunction, mean (SD)	17.4 (20.6)	17.3	17.5 (N = 744)	n.a.	n.a.
	Communication deficit, mean (SD)	14.0 (21.1)	35.8	17.5 (N = 742)	n.a.	n.a.
CFQ^f^		N = 205		N = 48	N = 30	N = 1358
	Total score, median (IQR)	20 (10-30)	n.a.	31.5	n.a.	n.a.
	Total score, mean (SD)	21.3 (14.0)	n.a.	n.a.	27.6	31.8

a EQ-5D VAS scores range from 0 to 100, with a higher score indicating better outcomes. Calculation is done according to the manual^80^ and then compared with the normative values of the healthy population,^27^ a cancer population including patients with different primary tumors^57^ and a glioblastoma population.^44^

b C30 scores range from 0 to 100, with a higher score for a functional scale representing a high/healthy level of functioning. A high score for a symptom scale/item represents a high level of symptomatology/problems. Calculation is done according to the manual^81^ and then compared with the normative values of the brain metastases population including patients with different primary tumors,^9^^,^^58^ the healthy Dutch population,^42^ cancer population including patients with different primary tumors,^41^ and brain tumor population including low- and high-grade subtypes.^58^

c HADS scores range from 0 to 21 for anxiety and 0 to 21 for depressions, with a higher score indicating more anxiety symptoms or depression symptoms. A score ≥8 indicates signs of anxiety or depression. Calculation is done according to the manual^82^ and then compared with the normative values of the healthy population,^48^ colorectal cancer population,^48^ and glioma population.^49^

d MFI total score ranges from 20 to 100. MFI subscale-scores range from 4 to 20, with a higher score indicating more fatigue. Calculation is done according to the manual^83^ and then compared with the normative values of the healthy population,^39^ cancer population including patients with different primary tumors,^45^ and low-grade glioma population.^40^

e The QLQ-BN20 consists of 11 multi-item scales of which we used four items which uses multiple questions that address: future uncertainty (4 questions); visual disorder (3 questions); motor dysfunction (3 questions); and communication deficit (3 questions). All items and scale scores of the QLQ-BN20 are linearly transformed to a 0-100 scale, with higher scores reflecting more severe symptoms. Calculation is done according to the manual^84^ and then compared with the normative values of the brain metastases population including lung and breast cancer^9^ and brain tumor population including highly anaplastic oligodendroglioma and newly diagnosed glioblastoma multiforme.^26^

f CFQ total score ranges from 0 to 100, with a higher score indicating more cognitive impairments. Calculation is done according to the manual^85^ and then compared with the normative values of the healthy population,^36^ breast cancer population,^50^ and lower grade gliomas.^47^

BMs = brain metastases; CFQ = cognitive failures questionnaire; HADS = hospital anxiety and depression scale; IQR = interquartile range; MFI = multidimensional fatigue inventory; n.a. = not available; SD = standard deviation; VAS = visual analogue scale.

COIMBRA patients reported a mean *EQ-5D VAS score* of 65.2. *C30* questionnaire results showed lower mean *global health status* compared to the healthy population, but higher than previously reported for the BMs population. Mean *social and emotional functioning scores* were higher, while *role* and *cognitive functioning scores* were lower compared to the in literature BMs population. *Financial problems* were reported less frequently than in the reference BMs and cancer populations.

The median *anxiety score* was 4 on the *HADS* scale, with 20.2% of respondents showing *signs of anxiety* (score ≥8), and the median *depression score* was 3, with 19.2% indicating *signs of depression* (score ≥8). COIMBRA patients also reported higher mean *total MFI scores* than all reference populations, indicating higher levels of fatigue.

*BN20 scores* highlighted specific neurological impairments. The mean *future uncertainty score* was higher than reported for the BM population in literature. COIMBRA patients reported a lower mean *communication deficit* and *visual disorder* compared to the BMs population. Mean *motor dysfunction scores* were similar for COIMBRA patients and the BMs population. Compared to the primary brain tumor population, COIMBRA patients had lower mean *future uncertainty score* and mean *communication deficit scores*, higher mean *visual disorder score*, and equal mean *motor dysfunction score*. Cognitive complaints, as measured by the *CFQ* questionnaire at baseline, were low compared to the literature on the other mentioned populations.

Following FDR correction, significant correlations were observed between *EQ5D-VAS* and most patient-related questionnaires, except for *anxiety* and *depression scores* measured by HADS and C30 *financial problems*. Positive for functional scales and negative for symptom scales. Financial issues did not show significant correlations with any QoL or functioning scales across the included questionnaires. *Depression scores* showed significant positive correlations with *fatigue* and significant negative correlations with most *C30 functional scales*. Anxiety showed significant positive correlations with *fatigue* and its subdomains but not with reduced activity. *CFQ total score* exhibited significant positive correlations with *total MFI score* and *mental fatigue,* and significant negative correlations with *emotional* and *cognitive functioning* measured in the C30. Supplementary Table 3A-C show the complete details of the patient QoL correlation analyses.

#### Caregiver QoL and Strain Index

Questionnaire return rate among caregivers was 77.4 % at baseline. Results of the COIMBRA caregiver questionnaires compared with those of healthy individuals, and caregivers of patients with BMs, cancer, and primary brain tumors, are shown in Table 3.

**Table 3.** COIMBRA—caregiver-reported outcomes compared to reference scores of healthy, cancer, and brain tumor populations.

Questionnaire	Domain/sub-score	COIMBRA caregivers	BMs caregivers	Primary brain tumor caregivers	General cancer caregivers	Healthy population
CSI^a^		N = 80		N = 14	N = 100	
	Total score, mean (SD)	4.9 (3.1)	n.a.	3.6	4.0	n.a.
	Signs of burden, %	30.0	n.a.	36.0	25.0	n.a
EQ-5D^b^		N = 82			N = 36	N = 2367
	VAS, median (IQR)	80 (75 - 90)	n.a.	n.a.	73	82
HADS^c^		N = 81	N = 21	N = 49	N = 31	N = 315
	Anxiety score, mean (SD)	6.2 (3.9)	7.48	10.6	8.2	3.5
	Depression score, mean (SD)	4.4 (3.3)	9.18	7.2	6.4	3.8
	Signs of anxiety, %	38.3	n.a	49.0	46.6 (N = not reported)	11.8
	Signs of depression, %	16.0	n.a	20.0	42.3 (N = not reported)	12.8

a CSI scores range from 0 to 13, with a higher score indicating more signs of burden. A score ≥7 indicates signs of burden. Calculation is done according to the manual^32^ and then compared with the normative values of the cancer population without specific tumor subtype specification^51^ and high-grade glioma population.^52^

b EQ-5DVAS scores range from 0 to 100, with a higher score indicating better outcomes. Calculation is done according to the manual^80^ and then compared with the normative values of the healthy population^27^ lung and upper gastrointestinal tract cancer population^76^ and multiple sclerosis as brain tumor data were not available.^53^

c HADS scores range from 0 to 21, with a higher score indicating more anxiety or depression. A score ≥8 indicates signs of anxiety or depression. Calculation is done according to the manual^82^ and then compared with the normative values of the brain metastases population including patients with different primary tumors.^15^ Healthy population, cancer population including patients with different primary tumors,^54^^,^^55^ and brain tumor population including glioblastoma multiforme and grade III and meningioma.^56^

BMs = brain metastases; CSI = caregiver strain index; HADS = Hospital Anxiety and Depression Scale; IQR = interquartile range; n.a. = not available; SD = standard deviation; VAS = visual analogue scale.

The median *CSI total score* was 5, with 30% of the respondents experiencing *signs of burden* (score ≥7). Median *EQ-5D VAS score* for caregivers was 80. *HADS scores* showed a median *anxiety score* of 6 and a median *depression score* of 5. Additionally, 38.3% of the caregivers reported *signs of anxiety* and 16.0% reported *signs of depression*.

After applying FDR correction, the *CSI total score* showed significant positive correlations with caregiver *anxiety* and *depression scores*, and a significant negative correlation with PROs in *physical functioning, global health, role functioning, and social functioning* domains within the *C30 scale*. Notably, caregiver *depression* and *anxiety* exhibited a moderate, significant positive correlation with each other, but did not significantly correlate with other variables, except for caregiver depression and *EQ5D-VAS* caregiver, which showed a significant negative correlation*. EQ5D-VAS* caregiver did not show significant correlations with PROs. Supplementary Table 3A-C show the complete details of the caregiver QoL and strain index correlation analyses.

## Discussion

BMs impact patients’ QoL and contribute to caregiver burden. Although BMs do not directly affect caregivers, their impact on caregiver burden is mediated indirectly through the patient’s clinical status and care requirements. Evaluating clinical characteristics and QoL pre-radiotherapy is necessary for informed treatment decisions and to support patients and caregivers.^10^^,^^12^ This article introduces the study setup and a sub-study of COIMBRA, which presents assessments for both patients and caregivers before stereotactic radiosurgery.

### Study Design

COIMBRA aligns with other programs in our institute, with the adage “learning from every patient”. Similar cohorts exist for breast cancer, colorectal cancer, and bone metastases, to study new interventions.^62–64^ Our cohort’s high consent rate (71.1%) for the TwiCs framework validates the feasibility of this approach and establishes a valuable platform for future clinical trials. The engagement rate facilitates rapid and efficient testing of novel interventions, potentially accelerating improvements in BMs management.

The questionnaire return rate in COIMBRA (78.3%) was lower than in bone metastases (90%)^65^ and the breast cancer cohorts (80%),^66^ possibly reflecting the higher disease burden among patients with BMs. Compared to other BM-specific studies (eg, Habets et al reporting 98% response in a selected, likely fitter cohort undergoing neuropsychological assessments)^67^ COIMBRA includes a broader spectrum of BMs patients, including those with lower KPS, therefore this likely makes our response rate a more realistic representation of the BMs population.

### Clinical Characteristics

Our cohort’s median age was 66, with 69.2% symptomatic BMs, predominantly from lung, breast, or melanoma primaries, which aligns with current literature.^1^^,^^68^ We observed a higher proportion of patients with single BM (48.3% vs the reported 34.4%^4^) suggesting a potential shift in disease presentation or detection methodology. The inclusion of patients with a KPS < 70 (8.5%) contrasts with current guidelines, generally advising against radiotherapy for this group.^3^ However, KPS may reflect neurological symptoms that respond to radiotherapy. This highlights the importance of individualized risk-benefit assessments to determine which patients with poor performance status may benefit from radiotherapy.

The spatial distribution of BMs in our study revealed distinct patterns with high-prevalence areas, or “hotspots”, identified in specific brain regions and white matter tracts. These findings suggest that BM localization is not random, but influenced by underlying anatomical and physiological factors. The observed predominance in the cerebellum, particularly the left cerebellar lobe, and in supratentorial areas such as the occipital lobes, right pre- and postcentral gyri, and specific frontal and temporal regions, along with involvement of critical white matter pathways, can have significant clinical implications such as symptoms or survival. Preliminary analyses suggest links between lesion location, disruption of critical white matter pathways, and neurological deficits measured by PROs. Lesions in regions like the cerebellum and motor cortex often cause specific symptoms such as coordination problems and motor deficits.^69^ Recognizing these specific areas of high lesion prevalence helps in predicting potential symptoms and complications, thereby aiding in more accurate clinical assessments.^70^ However, our cohort may be biased, as smaller solitary BM at accessible locations may be resected without subsequent radiotherapy, and thus excluded from our cohort. Therefore, our cohort may include relatively high amount of BMs located in challenging areas from a surgical perspective, such as the cerebellum or eloquent regions like the motor cortex.

### QoL-Patient

Our study provides extensive information on QoL of patients before SRS. We observed lower median QoL scores in our cohort compared to patients with a primary brain tumor, but higher than the general cancer population, and with fewer *signs of anxiety* and *depression*. However, our findings also highlighted higher mean *total score* for* fatigue*, *physical fatigue*, and *reduced motivation* compared to all other populations. These results underscore the complex QoL profile of patients with BMs and the need for personalized care.

The *EQ-5D VAS* and *C30* results revealed lower *median QoL scores* in our cohort compared to healthy individuals.^27^^,^^42^ Interestingly, our patients showed a higher mean *global health status* and *emotional functioning* on the *C30* compared to the cancer population.^41^^,^^57^ This could be attributed to the palliative care focus on symptom management and QoL, leading to better perceived general health.^13^ Additionally, patients with BMs may have adjusted their health expectations, resulting in more positive self-reporting.^10^^,^^13^ The differences in mean *C30 global health scores* between our study and previous research^58^ likely reflect methodological variations and diverse patient populations. QoL results must be interpreted within the context of each specific study and population.

Despite reporting higher mean *general health status* in the *C30*, our population showed lower mean scores in *physical-, role-, and social functioning* compared to patients with cancer of different primary tumors.^41^ This discrepancy is evident in the positive Pearson correlation coefficients between the subdomains of the *C30*. The specific challenges faced by patients with BMs appear to impact these domains more profoundly. This aligns with Meyers et al, who noted that patients with a brain tumor often experience impairments in physical and cognitive functioning, which can directly affect their role and social functioning.^71^ This suggests a disconnect between perceived overall health and their specific functional capabilities, suggesting that standard QoL measures may not fully capture the challenges faced by patients with BMs.

Our population reports higher mean *levels* of *fatigue* across all domains compared to healthy individuals and patients with cancer, aligning with their deteriorated overall health scores.^39^ We observed strong correlations between *MFI scores* and both *EQ-5D VAS* and *global health scores*, emphasizing the central role fatigue plays in patients’ overall well-being. Compared to the primary brain tumor population, our population shows higher mean *physical fatigue*, *reduced activity*, and *reduced motivation*, but lower mean *general fatigue* and *mental fatigue*.^40^ Given the pronounced impact of fatigue across multiple domains, its assessment and management should be considered during treatment planning and palliative care strategies.

In current study, patients reported lower mean *cognitive functioning* on the *C30* compared to healthy individuals and the cancer population, but higher than those with primary brain tumors. Interestingly, the cognitive complaints reported in the *C30* were not reflected to the same extent in the *CFQ* for our population. This discrepancy confirms the challenges of subjectively measuring cognitive function as already shown previously by Van Grinsven et al in a study on cognitive functioning of a subset of these COIMBRA patients.^72^ The significant positive correlation between *CFQ total score* and *fatigue scores* suggests a strong interplay between experienced *cognitive function* and *fatigue*. This interplay is further supported by the correlations between higher cognitive failure *CFQ scores* and lower *emotional/cognitive functioning* on the *C30*. These associations highlight the multifaceted nature of cognitive complaints in BM patients, influenced by and interconnected with symptoms such as fatigue and emotional distress. Patients reporting more cognitive complaints showed worse emotional and cognitive functioning and experienced more general or mental fatigue.

In our population, a lower percentage of patients reported *signs of anxiety* compared to cancer and primary brain tumor populations.^48^^,^^49^ This difference might be attributed to the longer duration of the patient journey in individuals with BMs, who are often diagnosed with metastases later in their disease trajectory. While not specifically studied in BM patients, research on chronic illnesses suggests that patients may develop more effective coping strategies over time.^73^ The concept of post-traumatic growth could also play a role in explaining the lower anxiety and depression rates compared to patients with a primary brain tumor,^74^ particularly considering that primary brain tumor patients are often surveyed closer to their initial diagnosis. This timing difference in assessment could contribute to the observed variations in psychological outcomes. Despite these relatively lower rates, anxiety and depression percentages in our cohort still exceeded those in the healthy population, emphasizing the importance of continued psychological support tailored to the unique needs of BM patients throughout their disease course.

Analysis of the *BN20* questionnaire showed higher mean *visual disorder* and lower mean *communication deficit* compared to the primary brain tumor population,^75^ which can be directly linked to the specific distribution of metastases observed in our study. The higher prevalence of visual disorders aligns with our finding of hotspots in both occipital lobes, which are crucial for visual processing. This distribution pattern explains the increased incidence of visual symptoms in our cohort. Mean *motor dysfunction* scores were comparable between the groups, highlighting shared challenges in mobility and physical coordination.

Our analysis revealed an interesting finding that *financial issues* did not show significant correlations with any *QoL* or *functioning scales* across the included questionnaires. This lack of correlation might be partly explained by the robust social support system of the Netherlands. The country has a universal health insurance system that covers most medical expenses, including treatments for serious conditions like BMs. Additionally, the Netherlands provides strong support for those facing unemployment or disability, which could mitigate financial stress for patients unable to work due to their condition.

### QoL-Caregiver

Our study provides valuable insights into the QoL and burden of caregivers for patients with BMs before SRS. Primarily, we can conclude that despite caregivers reporting relatively good overall health status, a notable proportion of caregivers showed *signs of anxiety* and *burden*, highlighting the substantial burden.

Caregivers of COIMBRA patients reported higher burden compared to caregivers of cancer and primary brain tumor patients.^51^^,^^52^ Paradoxically, they also reported higher *EQ-5D VAS scores* and lower *anxiety* and *depression rates*.^76^ This contradiction may be explained by the unique trajectory of BM patients and their caregivers. Unlike rapidly progressing primary brain tumors, BMs typically occur later in the cancer journey, allowing caregivers to develop resilience over time. This aligns with Sherwood et al’s findings on brain tumor caregivers’ resilience development.^77^ The extended caregiving period for BM patients may foster more robust coping mechanisms, resulting in higher perceived QoL and lower psychological distress despite ongoing challenges. These findings underscore the complex interplay between caregiver burden, adaptive coping, and the prolonged caregiving journey in BM cases, highlighting the need for targeted support interventions that recognize both challenges and resilience potential in this population.

The analysis revealed significant positive correlations between *CSI total score* and caregiver *anxiety*/*depression scores*, along with negative correlations between the *CSI* and the *physical-, role-*, and *social functioning* of the patient. These findings underscore relationship between patient and caregiver well-being in the BMs population. As patients experience declines in physical capabilities, ability to perform daily activities, and social interactions, caregivers tend to report higher levels of strain, anxiety, and depression. This interdependence echoes with previous findings by Boele et al, emphasizing the bidirectional impact of patient symptoms and caregiver burden in neuro-oncology settings.^78^

Our study has several limitations. First, a selection bias may arise because participation requires active consent and questionnaire completion. Patients with poorer health or higher symptom burden might be less able or willing to participate fully, which could underestimate the true burden. Second, missing and incomplete data remain a challenge. For example, we discontinued the SQUASH (Short QUestionnaire to ASsess Health-enhancing physical activity) due to low completion rates, as patients found it lengthy or confusing. This emphasizes the tradeoff between collecting comprehensive data and minimizing patient burden in a vulnerable population. Although we chose validated, concise instruments, balancing data richness, and feasibility remains difficult. Modern data collection systems could optimize questionnaire selection and completion, reducing patient burden while maintaining comprehensive data collection.^79^ Additionally, exploring innovative ways to engage patients who may be too unwell to complete extensive questionnaires remains difficult and affects representativeness. Additionally, the reference values used to PRO comparisons originate from studies including heterogeneous populations with varying tumor types and grades. For example, primary brain tumor cohorts include subtypes such as low-grade gliomas and glioblastomas, which differ substantially in prognosis, symptom burden, and QoL. This heterogeneity limits interpretation, as outcomes may vary across tumor subtypes.

### Clinical Implications and Recommendations

COIMBRA addresses significant unmet needs in BMs care, as highlighted by *National Cancer Institute Collaborative Workshop on Shaping the Landscape of Brain Metastases Research*.^12^ By systematically mapping patient and caregiver-reported outcomes before SRS, COIMBRA offers a comprehensive and pragmatic framework to improve understanding, management and research of this (complex) vulnerable population. This framework provides a practical reference for other institutions aiming to monitor and improve oncology care for patients and their families.

Clinically, integrating validated PROs such as the EORTC QLQ-C30 (including BN20), HADS, and MFI into routine practice at key treatment milestones (pre-SRS, and 1, 3, and 6 months post-treatment) enables systematic identification of under-recognized psychological and caregiver burdens. These questionnaires provide insights that support shared decision-making and highlight the urgent need for integrated psychosocial support as a standard component of oncology care. Given the significant burden experienced by caregivers, their structured assessment and targeted interventions should be prioritized.

Methodologically, the TwiCs-based COIMBRA infrastructure provides a valuable platform for longitudinal monitoring and pragmatic clinical trials designed to evaluate interventions that target symptoms and QoL. Integrating these tools to clinical care ensures large-scale feasibility and allows for faster translation of findings into daily practice.

Building on these foundations, COIMBRA also enables future research, including subgroup analyses survival comparisons and longitudinal imaging to study clinical and QoL outcomes. Advanced approaches such as brain connectivity,^17^ together with investigations of coping, personality, and caregiver resilience, can further refine tailored psychosocial interventions and improve patient- and caregiver-centered care.

### Conclusion

We designed the COIMBRA cohort and collected clinical and QoL data from a large sample of patients with BMs and their caregivers. This study highlights the distinct challenges faced by this patient population, including impairments in physical, emotional, and cognitive functioning, with high levels of fatigue. Patients reported similarly low QoL scores compared to those with primary brain tumors or other cancers, emphasizing the significant impact of their condition. Caregivers, despite reporting relatively good overall health, experience notable signs of anxiety and burden.

These findings emphasize the unmet needs of both patients and caregivers, indicating an urgent need for targeted support systems. They also highlight the importance of integrating patient- and caregiver-reported outcomes into routine care and policy planning, while laying the groundwork for future research on predictive factors for QoL, overall survival, and radiological changes after radiotherapy in patients with BMs.

## Supplementary Material

npaf105_Supplementary_Data

## Data Availability

Due to patient privacy concerns, data for this study cannot be shared with current article. Data sharing is possible from patients who agreed on sharing their data upon the establishment of a data sharing agreement between UMC Utrecht and interested parties.
