# Supplementary material for: Understanding the unmet needs of patients with brain metastases referred for stereotactic radiotherapy and their caregivers: A prospective cohort study
Source: Neurooncol Pract. 2025 Oct 7;13(2):262–75. doi: 10.1093/nop/npaf105 (PMC13153698; doi:10.1093/nop/npaf105)
Supplement: npaf105_Supplementary_Data [file npaf105_supplementary_data.docx]

## **9. Supplementary material**

| **Supplementary Text 1: Description of additional components of COIMBRA** |
| --- |

**Neurocognitive assessment**

In addition, starting June 2021, participants could choose to complete a comprehensive neurocognitive assessment (NCA) lasting a maximum of 90 minutes. This assessment evaluated the most commonly tested neurocognitive domains, wherein several domains are known to be vulnerable to impairment due to tumor- and (radio-)therapy-effects. The NCAs were administered before the start of radiotherapy, 3 months post-radiotherapy, and more than 11 months post-radiotherapy. For detailed information on the methodology and specific procedures used in the NCA, see Van Grinsven et al. (2023)^1^ and **Supplementary Table 2** for a summary of the neuropsychological tests administered per neurocognitive domain.

**Additional imaging**

As fourth option, a patient could consent for undergoing additional MRI sequences, which are not part of regular clinical imaging. The purpose of these extra sequences was to evaluate the feasibility, performance, and added value of new MRI techniques. These research sequences could be performed immediately following a regularly scheduled clinical MRI for consenting patients. No additional actions were required from the patient, with the only burden being the extra time needed for these sequences. The additional imaging could take up to 15 minutes, and to minimize patient burden, the total scan time (including both regular and experimental sequences) was limited to 60 minutes.

**Future randomization for TwiCs**

Patients were asked to consent to future randomization within TwiCs framework. When a new intervention is ready for evaluation, eligible patients within the observational cohort are identified. From this so-called sub-cohort, patients are randomized into either undergoing the intervention or receiving standard treatment. Only those selected for the intervention are invited to participate, requiring additional written informed consent. If patients decline, they will receive standard care, without being informed that they function as control group. This process is called staged informed consent: patients initially consent to undergo randomization for future studies, and at a later time consent to participate in the intervention arm of a new trial.^2^ Outcomes of the selected patients receiving the new intervention will be compared to the outcomes of the non-selected patients receiving standard treatment. Within this cohort, the same process can be simultaneously performed for other interventions (**see Supplementary Figure 1**).

**Sharing pseudonymized data with third parties**

From September 2022 data of patients could be shared with third parties if the patient explicitly agreed upon in the informed consent. Data can be shared only in pseudonymized form to third parties that comply with the European privacy regulation GDPR33 and that use secure transfer methods. Data could comprise images, clinical structure delineation, diagnosis, and treatment plan.

| **Supplementary Text 2: Details of the MRI Protocol and hotspot methodology** |
| --- |

MR images were acquired on 1.5T Philips Ingenia scanner (Philips Medical Systems, Best, The Netherlands) as on baseline and follow-up on 3T Philips as part of routine clinical care. T1-weighted MR images were acquired with a 3D turbo-spin echo (TSE) sequence with and without gadolinium enhancement voxel resolution 1.0 mm3. The planning CT scans were acquired on a Brilliance Big bore scanner (Philips Medical Systems, Best, The Netherlands), with a tube potential of 120 kVp, with use of a matrix size of 512 × 512 and 0.65 × 0.65 × 3.0 mm voxel size. Spatial normalization of the planning T1 MRIs to MNI152 space was performed with SynthMorph, a deep-learning-based, anatomy-aware, contrast and resolution agnostic tool for deformable image registration.^3^ After the registration was calculated, the very same transformation was applied to the GTVs to acquire the population-based lesion overview (‘hotspot’) map, which allows voxelwise assessment of the GTVs across all patients.

| Supplementary Table 1: Overview of patient-reported outcomes (PROs) and caregiver-reported outcomes questionnaires | | | | | |
| --- | --- | --- | --- | --- | --- |
| Questionnaire name | **Domain measured** | **Subdomains** | **Number of questions** | **Scoring methods** | **Interpretation** |
| EORTC QLQ-BN20^4^ | Brain-specific quality of life | Future uncertainty  Visual disorder  Motor dysfunction  Communication deficit  Headaches  Seizures  Drowsiness  Hair loss  Itchy skin  Weakness of legs  Bladder control. | 20 | 4-point Likert scale | Calculated scores range from 0 to 100 scale  High scores reflecting more severe symptoms |
| Caregiver strain index (CSI)^5^ | Caregiver strain | - | 13 | Yes/No | Calculated score range from 0 to 13  Higher score indicating more signs of burden  A score ≥ 7 indicates signs of burden |
| Cognitive failures questionnaire (CFQ)^6^ | Cognitive symptoms | Memory  Distractibility  Blunders  Forgetfulness | 25 | 5-point Likert scale | Calculated total score ranges from 0 to 100  High score indicating more cognitive impairment |
| EQ-5D-3L^7^ | General health status | VAS, median (IQR) | 5 (plus VAS) | 3-level scale plus visual analog scale | Calculated scores range from 0 to 100,  Higher score indicating better outcomes |
| EORTC QLQ-C30^8^ | General health-related quality of life | Global health status/QoL  Physical functioning  Role functioning  Emotional functioning  Cognitive functioning  Social functioning  Fatigue  Nausea and vomiting  Pain  Dyspnea  Insomnia  Appetite loss  Constipation  Diarrhea  Financial problems | 30 | Global health status: 7-point Likert scale  Other items: 4-point Likert scale | Calculated scores range from 0 to 100  High score for a functional scale representing a high / healthy level of functioning.  High score for a symptom scale / item represents a high level of symptomatology / problems. |
| Hospital anxiety and depression score (HADS)^9^ | Anxiety Depression | Anxiety  Depression | 14 | 4-point Likert scale | Calculated scores range from 0 to 21 for anxiety or depression  Higher score indicating more anxiety/depression symptoms.  A score ≥ 8 indicates signs of anxiety/depression. |
| Multifatigue index (MFI)^10^ | Fatigue | Total score  General fatigue score  Physical fatigue score  Reduced activity score  Reduced motivation  Mental fatigue score | 20 | 5-point Likert scale | Calculated total score ranges from 20 to 100, with a higher score indicating more fatigue  MFI subscale-scores range from 4 to 20, with a higher score indicating more fatigue |
| Neo-five factor inventory (NEO-FFI)^11^ | Personality traits | Neuroticism  Extroversion  Openness to experience Agreeableness Conscientiousness | 60 | 5-point Likert scale | For each dimension (scale), the scores of the corresponding questions are added together, then these scores are converted into stanines from “very low” to “very high.” Also an average score is displayed per dimension |
| Utrechtse coping lijst (UCL)^12^ | Coping style | Active approach  Palliative reaction, Avoidance  Social support  Passive reaction, Expressing emotions, Reassuring thoughts | 47 | 4-point Likert scale | Scores of items concerning the same coping strategy were summed to form a total score, with high scores indicating an increased tendency towards using that specific coping strategy |

This table details the name of each questionnaire, the primary domain measured, specific subdomains (if applicable), number of questions, scoring methods, and interpretation guidelines. This information provides context for understanding the various instruments used to evaluate quality of life and related outcomes in patients with brain metastases and their caregivers.

| Supplementary Table 2. Neuropsychological tests per neurocognitive domain. |
| --- |
| Premorbid intelligence |
| Ravens Advanced Progressive Matrices^13^ |
| Attention |
| Wechsler Adult Intelligence Scale (WAIS-IV) Digit Span *Forward Score* ^14^ |
| Trail Making Test (TMT) *Switching ratio* *B/A* ^15,16^ |
| Stroop/Delis Kaplan Executive Function System (DKEFS) *Switching ratio IV vs III* ^17^ |
| Executive functioning |
| WAIS-IV Digit Span *Backward Score* ^14^ |
| Letter fluency (3 letters) ^18,19^ |
| Stroop D’KEFS *Inhibition ratio III vs I* ^17^ |
| Memory |
| Hopkins Verbal Learning Test – Revised (HVLT-R) *Immediate, delayed, and recognition* ^20,21^ |
| Rey-Osterieth Complex Figure Test (ROCFT) *Delayed copy* ^22^ |
| Visual Association Test (VAT) long form *Immediate and delayed* ^23^ |
| Semantic fluency ^24^ |
| Processing speed |
| TMT *A* ^15^ |
| Stroop/DKEFS *naming [I] and reading [II]* ^17^ |
| Psychomotor speed |
| Lafayette Grooved Pegboard *Dominant and non-dominant hand* ^25^ |
| Visuospatial functioning |
| ROCFT *direct copy* ^22^ |
| Hooper Visual Organization Test (HVOT) Fragmented ^26^ |
| Social cognition |
| Facial Expressions of Emotion – Stimuli and Tests (FEEST) Total score ^27^ |
| Language |
| HVOT non-fragmented [clinical interpretation] ^26,28^ |

This table details the performed neuropsychological test as part of COIMBRA


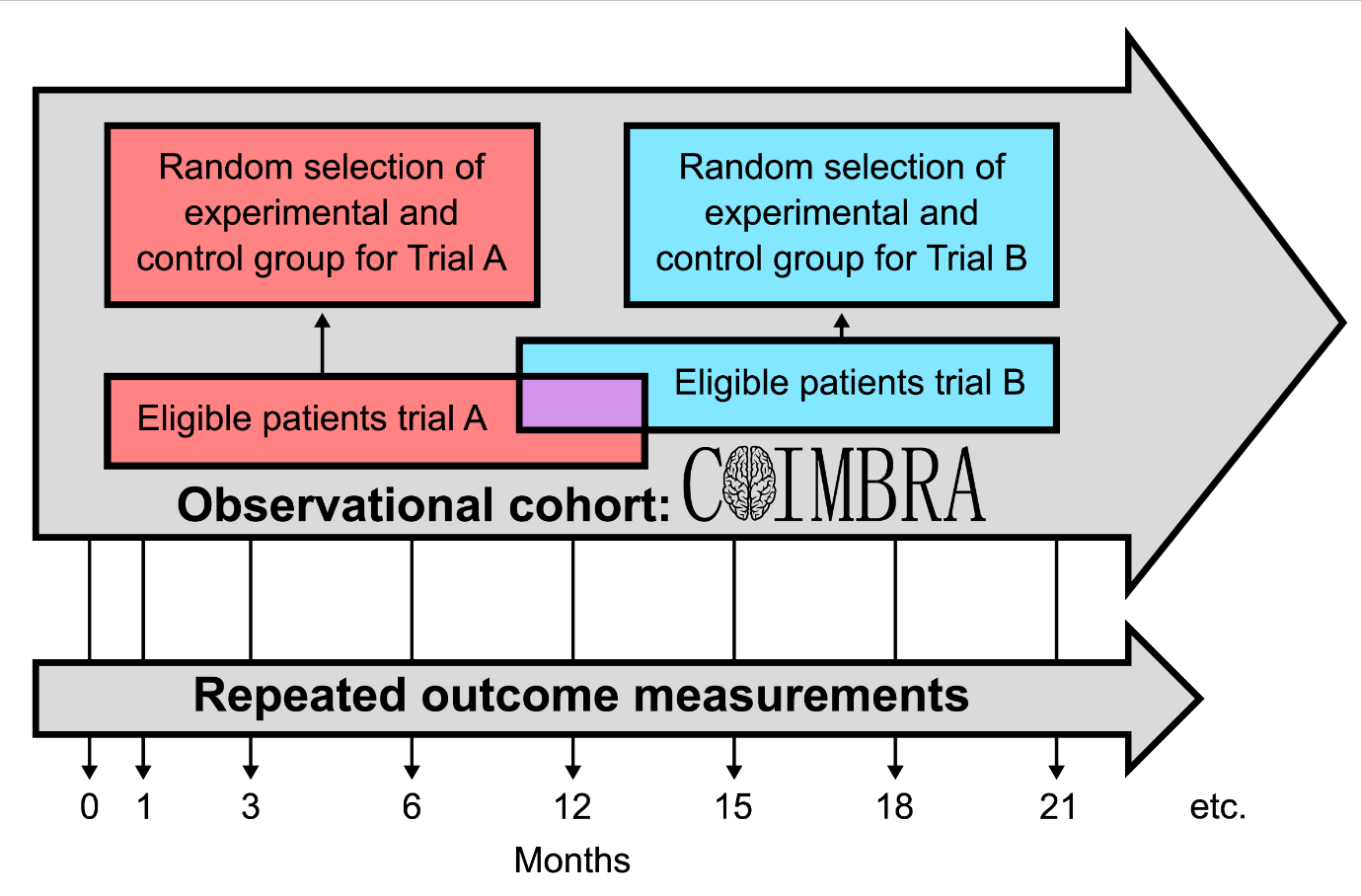


**Supplementary Figure 1: The Trials within Cohorts (TwiCs) design.** *A large observational cohort with the condition of interest is recruited. Participants’ clinical and self-reported outcomes are regularly measured. For each randomized trial a sub-cohort of eligible patient is identified. Patients from the sub-cohort are randomly selected to undergo the intervention. Their outcomes are then compared to the other patients in the sub-cohort who undergo standard care. This process repeats for each new TwiC that is conducted; patients may be eligible for multiple trials. Figure adapted from Relton et al. 2010.* ^29^

| **Supplementary table 3A:** Pearson correlation output between questionnaires | | | | | | | | | | | | | | | | | | | | | |
| --- | --- | --- | --- | --- | --- | --- | --- | --- | --- | --- | --- | --- | --- | --- | --- | --- | --- | --- | --- | --- | --- |
|  | **EQ5D-VAS patient** | **CSI total score** | **Anxiety patient** | **Depression patient** | **Anxiety caregiver** | **Depression caregiver** | **CFQ total score** | **MFI total score** | **MFI- general fatigue** | **MFI- physical fatigue** | **MFI-reduced activity** | **MFI-reduced motivation** | **MFI-mental fatigue** | **EQ5D- VAS caregiver** | **C30 -physical functioning** | **C30- global health status** | **C30 -role functioning** | **C30 – emotional functioning** | **C30 – cognitive functioning** | **C30 – social functioning** | **C30- financial problems** |
| **EQ5D-VAS patient** | 1 | -0.223 | -0.239 | -0.492 | -0.037 | -0.151 | -0.139 | -0.586 | -0.481 | -0.585 | -0.512 | -0.500 | -0.299 | 0.109 | 0.527 | 0.724 | 0.515 | 0.304 | 0.347 | 0.448 | -0.065 |
| **CSI total score** | -0.223 | 1 | 0.283 | 0.359 | 0.422 | 0.487 | -0.068 | 0.258 | 0.291 | 0.246 | 0.288 | 0.244 | 0.160 | -0.249 | -0.395 | -0.484 | -0.397 | -0.339 | -0.266 | -0.461 | 0.339 |
| **Anxiety patient** | -0.239 | 0.283 | 1 | 0.600 | 0.179 | 0.041 | 0.394 | 0.384 | 0.281 | 0.253 | 0.193 | 0.379 | 0.458 | 0.083 | -0.045 | -0.249 | -0.182 | -0.703 | -0.358 | -0.295 | 0.157 |
| **Depression patient** | -0.492 | 0.359 | 0.600 | 1 | 0.130 | 0.269 | 0.266 | 0.651 | 0.517 | 0.515 | 0.509 | 0.703 | 0.436 | -0.116 | -0.350 | -0.548 | -0.337 | -0.587 | -0.348 | -0.435 | 0.162 |
| **Anxiety caregiver** | -0.037 | 0.422 | 0.179 | 0.130 | 1 | 0.641 | 0.120 | 0.064 | 0.000 | -0.020 | 0.066 | 0.118 | 0.035 | -0.276 | -0.023 | -0.046 | 0.005 | -0.148 | -0.077 | -0.215 | 0.127 |
| **Depression caregiver** | -0.151 | 0.487 | 0.041 | 0.269 | 0.641 | 1 | -0.048 | 0.133 | 0.167 | 0.161 | 0.184 | 0.184 | 0.099 | -0.525 | -0.324 | -0.283 | -0.184 | -0.118 | -0.116 | -0.275 | 0.291 |
| **CFQ total score** | -0.139 | -0.068 | 0.394 | 0.266 | 0.120 | -0.048 | 1 | 0.296 | 0.161 | 0.133 | 0.095 | 0.201 | 0.581 | -0.115 | -0.050 | -0.124 | -0.087 | -0.291 | -0.510 | -0.209 | 0.123 |
| **MFI total score** | -0.586 | 0.258 | 0.384 | 0.651 | 0.064 | 0.133 | 0.296 | 1 | 0.869 | 0.887 | 0.864 | 0.822 | 0.621 | -0.045 | -0.645 | -0.686 | -0.560 | -0.390 | -0.418 | -0.443 | 0.179 |
| **MFI- general fatigue** | -0.481 | 0.291 | 0.281 | 0.517 | 0.000 | 0.167 | 0.161 | 0.869 | 1 | 0.831 | 0.670 | 0.583 | 0.418 | -0.037 | -0.558 | -0.579 | -0.493 | -0.280 | -0.341 | -0.363 | 0.219 |
| **MFI- physical fatigue** | -0.585 | 0.246 | 0.253 | 0.515 | -0.020 | 0.161 | 0.133 | 0.887 | 0.831 | 1 | 0.735 | 0.620 | 0.368 | -0.041 | -0.686 | -0.680 | -0.557 | -0.284 | -0.284 | -0.409 | 0.180 |
| **MFI-reduced activity** | -0.512 | 0.288 | 0.193 | 0.509 | 0.066 | 0.184 | 0.095 | 0.864 | 0.670 | 0.735 | 1 | 0.700 | 0.400 | -0.076 | -0.630 | -0.593 | -0.532 | -0.219 | -0.296 | -0.413 | 0.107 |
| **MFI- reduced motivation** | -0.500 | 0.244 | 0.379 | 0.703 | 0.118 | 0.184 | 0.201 | 0.822 | 0.583 | 0.620 | 0.700 | 1 | 0.473 | -0.032 | -0.461 | -0.555 | -0.361 | -0.423 | -0.251 | -0.347 | 0.113 |
| **MFI-mental fatigue** | -0.299 | 0.160 | 0.458 | 0.436 | 0.035 | 0.099 | 0.581 | 0.621 | 0.418 | 0.368 | 0.400 | 0.473 | 1 | -0.235 | -0.264 | -0.372 | -0.338 | -0.374 | -0.633 | -0.268 | 0.182 |
| **EQ5D- VAS caregiver** | 0.109 | -0.249 | 0.083 | -0.116 | -0.276 | -0.525 | -0.115 | -0.045 | -0.037 | -0.041 | -0.076 | -0.032 | -0.235 | 1 | 0.266 | 0.198 | 0.148 | -0.031 | 0.191 | 0.220 | -0.194 |
| **C30 - physical functioning** | 0.527 | -0.395 | -0.045 | -0.350 | -0.023 | -0.324 | -0.050 | -0.645 | -0.558 | -0.686 | -0.630 | -0.461 | -0.264 | 0.266 | 1 | 0.597 | 0.630 | 0.119 | 0.285 | 0.457 | -0.142 |
| **C30 – global health status** | 0.724 | -0.484 | -0.249 | -0.548 | -0.046 | -0.283 | -0.124 | -0.686 | -0.579 | -0.680 | -0.593 | -0.555 | -0.372 | 0.198 | 0.597 | 1 | 0.608 | 0.347 | 0.395 | 0.578 | -0.212 |
| **C30 – role functioning** | 0.515 | -0.397 | -0.182 | -0.337 | 0.005 | -0.184 | -0.087 | -0.560 | -0.493 | -0.557 | -0.532 | -0.361 | -0.338 | 0.148 | 0.630 | 0.608 | 1 | 0.272 | 0.396 | 0.608 | -0.136 |
| **C30 – emotional functioning** | 0.304 | -0.339 | -0.703 | -0.587 | -0.148 | -0.118 | -0.291 | -0.390 | -0.280 | -0.284 | -0.219 | -0.423 | -0.374 | -0.031 | 0.119 | 0.347 | 0.272 | 1 | 0.438 | 0.387 | -0.125 |
| **C30- cognitive functioning** | 0.347 | -0.266 | -0.358 | -0.348 | -0.077 | -0.116 | -0.510 | -0.418 | -0.341 | -0.284 | -0.296 | -0.251 | -0.633 | 0.191 | 0.285 | 0.395 | 0.396 | 0.438 | 1 | 0.446 | -0.157 |
| **C30 - social functioning** | 0.448 | -0.461 | -0.295 | -0.435 | -0.215 | -0.275 | -0.209 | -0.443 | -0.363 | -0.409 | -0.413 | -0.347 | -0.268 | 0.220 | 0.457 | 0.578 | 0.608 | 0.387 | 0.446 | 1 | -0.179 |
| **C30- financial problems** | -0.065 | 0.339 | 0.157 | 0.162 | 0.127 | 0.291 | 0.123 | 0.179 | 0.219 | 0.180 | 0.107 | 0.113 | 0.182 | -0.194 | -0.142 | -0.212 | -0.136 | -0.125 | -0.157 | -0.179 | 1 |
| Visual overview of the correlation coefficient for all total scores and subdomain of all used questionnaires. Red colors indicate significant negative correlations. Green colors indicate significant positive correlations. Yellow colors indicate nonsignificant correlations. Significance levels are determined after False Discovery Rate (FDR) correction.^30^  *BMs= brain metastases; CFQ = Cognitive Failures Questionnaire; HADS = Hospital Anxiety and Depression Scale; IQR = Interquartile range; MFI = Multidimensional Fatigue Inventory; n.a. = not available; SD = standard deviation; VAS = Visual Analogue Scale;* | | | | | | | | | | | | | | | | | | | | | |

| **Supplementary table 3B:** P-values for Pearson Correlations Between Questionnaires | | | | | | | | | | | | | | | | | | | | | |
| --- | --- | --- | --- | --- | --- | --- | --- | --- | --- | --- | --- | --- | --- | --- | --- | --- | --- | --- | --- | --- | --- |
|  | **EQ5D-VAS patient** | **CSI total score** | **Anxiety patient** | **Depression patient** | **Anxiety caregiver** | **Depression caregiver** | **CFQ total score** | **MFI total score** | **MFI- general fatigue** | **MFI- physical fatigue** | **MFI-reduced activity** | **MFI-reduced motivation** | **MFI-mental fatigue** | **EQ5D- VAS caregiver** | **C30 -physical functioning** | **C30- global health status** | **C30 -role functioning** | **C30 – emotional functioning** | **C30 – cognitive functioning** | **C30 – social functioning** | **C30- financial problems** |
| **EQ5D-VAS patient** |  | 4.66E-02 | 5.56E-04 | 5.38E-14 | 7.46E-01 | 1.77E-01 | 4.79E-02 | 1.22E-19 | 2.09E-13 | 2.16E-20 | 3.07E-15 | 3.37E-14 | 1.25E-05 | 3.32E-01 | 1.08E-15 | 2.48E-35 | 1.17E-15 | 7.82E-06 | 2.40E-07 | 8.37E-12 | 3.46E-01 |
| **CSI total score** | 4.66E-02 |  | 1.19E-02 | 1.16E-03 | 1.20E-04 | 5.37E-06 | 5.61E-01 | 2.52E-02 | 1.03E-02 | 3.08E-02 | 1.10E-02 | 3.37E-02 | 1.67E-01 | 2.66E-02 | 4.07E-04 | 6.15E-06 | 2.88E-04 | 2.25E-03 | 1.78E-02 | 1.89E-05 | 2.43E-03 |
| **Anxiety patient** | 5.56E-04 | 1.19E-02 |  | 1.08E-21 | 1.15E-01 | 7.18E-01 | 6.88E-09 | 2.53E-08 | 4.18E-05 | 2.47E-04 | 5.52E-03 | 2.91E-08 | 5.33E-12 | 4.65E-01 | 5.27E-01 | 3.07E-04 | 8.31E-03 | 4.12E-32 | 1.09E-07 | 1.59E-05 | 2.44E-02 |
| **Depression patient** | 5.38E-14 | 1.16E-03 | 1.08E-21 |  | 2.51E-01 | 1.53E-02 | 1.28E-04 | 2.51E-25 | 1.26E-15 | 1.82E-15 | 4.13E-15 | 1.37E-31 | 4.98E-11 | 3.03E-01 | 3.54E-07 | 1.13E-17 | 5.47E-07 | 1.05E-20 | 2.18E-07 | 4.81E-11 | 1.94E-02 |
| **Anxiety caregiver** | 7.46E-01 | 1.20E-04 | 1.15E-01 | 2.51E-01 |  | 1.19E-10 | 2.96E-01 | 5.82E-01 | 9.98E-01 | 8.64E-01 | 5.68E-01 | 3.05E-01 | 7.64E-01 | 1.31E-02 | 8.45E-01 | 6.84E-01 | 9.68E-01 | 1.91E-01 | 4.96E-01 | 5.57E-02 | 2.66E-01 |
| **Depression caregiver** | 1.77E-01 | 5.37E-06 | 7.18E-01 | 1.53E-02 | 1.19E-10 |  | 6.76E-01 | 2.48E-01 | 1.40E-01 | 1.57E-01 | 1.04E-01 | 1.06E-01 | 3.88E-01 | 4.99E-07 | 3.75E-03 | 1.08E-02 | 1.00E-01 | 2.96E-01 | 3.02E-01 | 1.31E-02 | 8.76E-03 |
| **CFQ total score** | 4.79E-02 | 5.61E-01 | 6.88E-09 | 1.28E-04 | 2.96E-01 | 6.76E-01 |  | 2.98E-05 | 2.18E-02 | 5.99E-02 | 1.81E-01 | 4.69E-03 | 1.41E-19 | 3.18E-01 | 4.92E-01 | 7.94E-02 | 2.16E-01 | 2.55E-05 | 6.24E-15 | 2.72E-03 | 8.16E-02 |
| **MFI total score** | 1.22E-19 | 2.52E-02 | 2.53E-08 | 2.51E-25 | 5.82E-01 | 2.48E-01 | 2.98E-05 |  | 6.26E-63 | 3.33E-69 | 1.34E-61 | 6.73E-51 | 6.07E-23 | 6.99E-01 | 7.51E-24 | 5.02E-29 | 4.34E-18 | 1.28E-08 | 6.47E-10 | 4.95E-11 | 1.11E-02 |
| **MFI- general fatigue** | 2.09E-13 | 1.03E-02 | 4.18E-05 | 1.26E-15 | 9.98E-01 | 1.40E-01 | 2.18E-02 | 6.26E-63 |  | 7.91E-55 | 1.01E-28 | 3.49E-20 | 2.89E-10 | 7.44E-01 | 8.68E-18 | 5.20E-20 | 2.46E-14 | 4.19E-05 | 4.14E-07 | 6.84E-08 | 1.47E-03 |
| **MFI- physical fatigue** | 2.16E-20 | 3.08E-02 | 2.47E-04 | 1.82E-15 | 8.64E-01 | 1.57E-01 | 5.99E-02 | 3.33E-69 | 7.91E-55 |  | 6.59E-37 | 3.40E-23 | 4.41E-08 | 7.23E-01 | 3.59E-29 | 1.35E-29 | 1.33E-18 | 3.18E-05 | 2.93E-05 | 7.69E-10 | 9.11E-03 |
| **MFI-reduced activity** | 3.07E-15 | 1.10E-02 | 5.52E-03 | 4.13E-15 | 5.68E-01 | 1.04E-01 | 1.81E-01 | 1.34E-61 | 1.01E-28 | 6.59E-37 |  | 1.65E-31 | 1.96E-09 | 5.06E-01 | 1.52E-23 | 3.82E-21 | 8.44E-17 | 1.44E-03 | 1.27E-05 | 5.19E-10 | 1.23E-01 |
| **MFI reduced motivation** | 3.37E-14 | 3.37E-02 | 2.91E-08 | 1.37E-31 | 3.05E-01 | 1.06E-01 | 4.69E-03 | 6.73E-51 | 3.49E-20 | 3.40E-23 | 1.65E-31 |  | 8.68E-13 | 7.83E-01 | 1.13E-11 | 9.17E-18 | 9.50E-08 | 3.25E-10 | 2.87E-04 | 3.83E-07 | 1.09E-01 |
| **MFI-mental fatigue** | 1.25E-05 | 1.67E-01 | 5.33E-12 | 4.98E-11 | 7.64E-01 | 3.88E-01 | 1.41E-19 | 6.07E-23 | 2.89E-10 | 4.41E-08 | 1.96E-09 | 8.68E-13 |  | 3.84E-02 | 1.63E-04 | 3.30E-08 | 5.44E-07 | 2.88E-08 | 7.65E-25 | 9.05E-05 | 8.60E-03 |
| **EQ5D- VAS caregiver** | 3.32E-01 | 2.66E-02 | 4.65E-01 | 3.03E-01 | 1.31E-02 | 4.99E-07 | 3.18E-01 | 6.99E-01 | 7.44E-01 | 7.23E-01 | 5.06E-01 | 7.83E-01 | 3.84E-02 |  | 1.88E-02 | 7.83E-02 | 1.87E-01 | 7.87E-01 | 8.84E-02 | 4.82E-02 | 8.54E-02 |
| **C30 - physical functioning** | 1.08E-15 | 4.07E-04 | 5.27E-01 | 3.54E-07 | 8.45E-01 | 3.75E-03 | 4.92E-01 | 7.51E-24 | 8.68E-18 | 3.59E-29 | 1.52E-23 | 1.13E-11 | 1.63E-04 | 1.88E-02 |  | 6.46E-21 | 5.79E-24 | 9.09E-02 | 3.63E-05 | 7.58E-12 | 4.35E-02 |
| **C30 – global health status** | 2.48E-35 | 6.15E-06 | 3.07E-04 | 1.13E-17 | 6.84E-01 | 1.08E-02 | 7.94E-02 | 5.02E-29 | 5.20E-20 | 1.35E-29 | 3.82E-21 | 9.17E-18 | 3.30E-08 | 7.83E-02 | 6.46E-21 |  | 7.35E-23 | 2.23E-07 | 2.24E-09 | 2.51E-20 | 1.91E-03 |
| **C30 – role functioning** | 1.17E-15 | 2.88E-04 | 8.31E-03 | 5.47E-07 | 9.68E-01 | 1.00E-01 | 2.16E-01 | 4.34E-18 | 2.46E-14 | 1.33E-18 | 8.44E-17 | 9.50E-08 | 5.44E-07 | 1.87E-01 | 5.79E-24 | 7.35E-23 |  | 6.06E-05 | 1.87E-09 | 6.81E-23 | 4.72E-02 |
| **C30 – emotional functioning** | 7.82E-06 | 2.25E-03 | 4.12E-32 | 1.05E-20 | 1.91E-01 | 2.96E-01 | 2.55E-05 | 1.28E-08 | 4.19E-05 | 3.18E-05 | 1.44E-03 | 3.25E-10 | 2.88E-08 | 7.87E-01 | 9.09E-02 | 2.23E-07 | 6.06E-05 |  | 2.16E-11 | 5.41E-09 | 7.03E-02 |
| **C30- cognitive functioning** | 2.40E-07 | 1.78E-02 | 1.09E-07 | 2.18E-07 | 4.96E-01 | 3.02E-01 | 6.24E-15 | 6.47E-10 | 4.14E-07 | 2.93E-05 | 1.27E-05 | 2.87E-04 | 7.65E-25 | 8.84E-02 | 3.63E-05 | 2.24E-09 | 1.87E-09 | 2.16E-11 |  | 7.27E-12 | 2.21E-02 |
| **C30 - social functioning** | 8.37E-12 | 1.89E-05 | 1.59E-05 | 4.81E-11 | 5.57E-02 | 1.31E-02 | 2.72E-03 | 4.95E-11 | 6.84E-08 | 7.69E-10 | 5.19E-10 | 3.83E-07 | 9.05E-05 | 4.82E-02 | 7.58E-12 | 2.51E-20 | 6.81E-23 | 5.41E-09 | 7.27E-12 |  | 8.71E-03 |
| **C30- financial problems** | 3.46E-01 | 2.43E-03 | 2.44E-02 | 1.94E-02 | 2.66E-01 | 8.76E-03 | 8.16E-02 | 1.11E-02 | 1.47E-03 | 9.11E-03 | 1.23E-01 | 1.09E-01 | 8.60E-03 | 8.54E-02 | 4.35E-02 | 1.91E-03 | 4.72E-02 | 7.03E-02 | 2.21E-02 | 8.71E-03 |  |
| Visual overview of the correlation coefficient for all total scores and subdomain of all used questionnaires. Red colors indicate significant negative correlations. Green colors indicate significant positive correlations. Yellow colors indicate nonsignificant correlations. Significance levels are determined after False Discovery Rate (FDR) correction.^30^  *BMs= brain metastases; CFQ = Cognitive Failures Questionnaire; HADS = Hospital Anxiety and Depression Scale; IQR = Interquartile range; MFI = Multidimensional Fatigue Inventory; n.a. = not available; SD = standard deviation; VAS = Visual Analogue Scale;* | | | | | | | | | | | | | | | | | | | | | |

| **Supplementary table 3C:** Sample Sizes for Pearson Correlations Between Questionnaires | | | | | | | | | | | | | | | | | | | | | |
| --- | --- | --- | --- | --- | --- | --- | --- | --- | --- | --- | --- | --- | --- | --- | --- | --- | --- | --- | --- | --- | --- |
|  | **EQ5D-VAS patient** | **CSI total score** | **Anxiety patient** | **Depression patient** | **Anxiety caregiver** | **Depression caregiver** | **CFQ total score** | **MFI total score** | **MFI- general fatigue** | **MFI- physical fatigue** | **MFI-reduced activity** | **MFI-reduced motivation** | **MFI-mental fatigue** | **EQ5D- VAS caregiver** | **C30 -physical functioning** | **C30- global health status** | **C30 -role functioning** | **C30 – emotional functioning** | **C30 – cognitive functioning** | **C30 – social functioning** | **C30- financial problems** |
| **EQ5D-VAS patient** | 212 | 80 | 205 | 207 | 80 | 81 | 203 | 198 | 207 | 207 | 207 | 202 | 206 | 81 | 200 | 210 | 211 | 209 | 211 | 211 | 210 |
| **CSI total score** | 80 | 80 | 78 | 79 | 78 | 79 | 76 | 75 | 77 | 77 | 77 | 76 | 76 | 79 | 76 | 79 | 79 | 79 | 79 | 79 | 78 |
| **Anxiety patient** | 205 | 78 | 209 | 208 | 79 | 80 | 201 | 197 | 206 | 206 | 206 | 201 | 205 | 80 | 198 | 206 | 209 | 207 | 208 | 207 | 206 |
| **Depression patient** | 207 | 79 | 208 | 211 | 80 | 81 | 203 | 199 | 208 | 208 | 208 | 203 | 207 | 81 | 201 | 208 | 211 | 209 | 210 | 209 | 208 |
| **Anxiety caregiver** | 80 | 78 | 79 | 80 | 81 | 81 | 78 | 76 | 78 | 78 | 78 | 77 | 77 | 80 | 77 | 79 | 80 | 80 | 80 | 80 | 79 |
| **Depression caregiver** | 81 | 79 | 80 | 81 | 81 | 82 | 79 | 77 | 79 | 79 | 79 | 78 | 78 | 81 | 78 | 80 | 81 | 81 | 81 | 81 | 80 |
| **CFQ total score** | 203 | 76 | 201 | 203 | 78 | 79 | 205 | 193 | 202 | 202 | 202 | 197 | 201 | 78 | 195 | 202 | 205 | 203 | 204 | 204 | 203 |
| **MFI total score** | 198 | 75 | 197 | 199 | 76 | 77 | 193 | 202 | 202 | 202 | 202 | 202 | 202 | 77 | 191 | 199 | 202 | 199 | 201 | 200 | 200 |
| **MFI- general fatigue** | 207 | 77 | 206 | 208 | 78 | 79 | 202 | 202 | 211 | 210 | 210 | 206 | 209 | 79 | 200 | 208 | 211 | 208 | 210 | 209 | 209 |
| **MFI- physical fatigue** | 207 | 77 | 206 | 208 | 78 | 79 | 202 | 202 | 210 | 211 | 210 | 205 | 209 | 79 | 200 | 208 | 211 | 208 | 210 | 209 | 209 |
| **MFI-reduced activity** | 207 | 77 | 206 | 208 | 78 | 79 | 202 | 202 | 210 | 210 | 211 | 205 | 209 | 79 | 200 | 208 | 211 | 208 | 210 | 209 | 209 |
| **MFI-reduced motivation** | 202 | 76 | 201 | 203 | 77 | 78 | 197 | 202 | 206 | 205 | 205 | 206 | 204 | 78 | 195 | 203 | 206 | 203 | 205 | 204 | 204 |
| **MFI-mental fatigue** | 206 | 76 | 205 | 207 | 77 | 78 | 201 | 202 | 209 | 209 | 209 | 204 | 210 | 78 | 199 | 207 | 210 | 207 | 209 | 208 | 208 |
| **EQ5D- VAS caregiver** | 81 | 79 | 80 | 81 | 80 | 81 | 78 | 77 | 79 | 79 | 79 | 78 | 78 | 82 | 78 | 80 | 81 | 81 | 81 | 81 | 80 |
| **C30 - physical functioning** | 200 | 76 | 198 | 201 | 77 | 78 | 195 | 191 | 200 | 200 | 200 | 195 | 199 | 78 | 204 | 202 | 204 | 202 | 204 | 203 | 202 |
| **C30 – global health status** | 210 | 79 | 206 | 208 | 79 | 80 | 202 | 199 | 208 | 208 | 208 | 203 | 207 | 80 | 202 | 213 | 212 | 211 | 213 | 212 | 211 |
| **C30 – role functioning** | 211 | 79 | 209 | 211 | 80 | 81 | 205 | 202 | 211 | 211 | 211 | 206 | 210 | 81 | 204 | 212 | 215 | 212 | 214 | 213 | 212 |
| **C30 – emotional functioning** | 209 | 79 | 207 | 209 | 80 | 81 | 203 | 199 | 208 | 208 | 208 | 203 | 207 | 81 | 202 | 211 | 212 | 213 | 213 | 212 | 211 |
| **C30- cognitive functioning** | 211 | 79 | 208 | 210 | 80 | 81 | 204 | 201 | 210 | 210 | 210 | 205 | 209 | 81 | 204 | 213 | 214 | 213 | 215 | 214 | 213 |
| **C30 - social functioning** | 211 | 79 | 207 | 209 | 80 | 81 | 204 | 200 | 209 | 209 | 209 | 204 | 208 | 81 | 203 | 212 | 213 | 212 | 214 | 214 | 213 |
| **C30- financial problems** | 210 | 78 | 206 | 208 | 79 | 80 | 203 | 200 | 209 | 209 | 209 | 204 | 208 | 80 | 202 | 211 | 212 | 211 | 213 | 213 | 213 |
| Visual overview of the correlation coefficient for all total scores and subdomain of all used questionnaires. Red colors indicate significant negative correlations. Green colors indicate significant positive correlations. Yellow colors indicate nonsignificant correlations. Significance levels are determined after False Discovery Rate (FDR) correction.^30^  *BMs= brain metastases; CFQ = Cognitive Failures Questionnaire; HADS = Hospital Anxiety and Depression Scale; IQR = Interquartile range; MFI = Multidimensional Fatigue Inventory; n.a. = not available; SD = standard deviation; VAS = Visual Analogue Scale;* | | | | | | | | | | | | | | | | | | | | | |

## **Supplementary references**

1. van Grinsven EE, Cialdella F, Verhoeff JJC, Philippens MEP, van Zandvoort MJE. Different profiles of neurocognitive functioning in patients with brain metastases prior to brain radiotherapy. Psychooncology. 2023 Oct 3;

2. Verweij ME, Gal R, Burbach JPM, Young-Afat DA, van der Velden JM, van der Graaf R, et al. Most patients reported positively or neutrally of having served as controls in the trials within cohorts design. J Clin Epidemiol. 2022 Aug;148:39–47.

3. Hoffmann M, Hoopes A, Greve DN, Fischl B, Dalca A V. Anatomy-aware and acquisition-agnostic joint registration with SynthMorph. Imaging Neuroscience. 2024 Jun 25;2:1–33.

4. R/scoring.QLQBN20.R [Internet]. [cited 2024 Jun 27]. Available from: https://rdrr.io/cran/QoLR/man/scoring.QLQBN20.html

5. MJH Jungen, A Krispin. Uitgebreide toelichting van het meetinstrument Caregiver Strain Index (CSI). ML Bokhorst. 2022 Mar;

6. Merkelbach H PR. Uitgebreide toelichting van het meetinstrument Cognitive Failure Questionnaire (CFQ) [Internet]. 2018. Available from: www.meetinstrumentenzorg.nl

7. EuroQol Group. Uitgebreide toelichting van het meetinstrumenten EuroQol 5D (EQ-5D) [Internet]. 2019. Available from: https://euroqol.org/publications/user-guides/

8. Fayers PM, European Organization for Research on Treatment of Cancer Study Group on Quality of Life. EORTC QLQ-C30 scoring manual. 2001.

9. Pouwer F SF van der PH. Uitgebreide toelichting van het meetinstrument Hospital Anxiety and Depression Scale (HADS) [Internet]. 1997. Available from: www.gl-assessment.co.uk

10. Smets EM. Uitgebreide toelichting van het meetinstrument Multidimensionele Vermoeidheids Index (MVI-20) [Internet]. 2020 [cited 2024 May 16]. Available from: www.meetinstrumentenzorg.nl

11. McCrae RR,. The NEO-PI/NEO-FFI manual supplement. Psychological Assessment Resources. 1989;

12. Sanderman R, Ormel J. De Utrechtse Coping Lijst (UCL): validiteit en betrouwbaarheid. Tijdschrift voor Psychologie en Gezondheid. 1992;32–7.

13. John Hugh Court, Raven J. Manual for Raven’s Progressive Matrices and Vocabulary Scales. Section 7, Research and References: Summaries of Published Normative Studies, Reliability Studies, Validity Studies, References to All Sections of the Manual. Oxford Psychologists. 1995;

14. Wechsler Adult Intelligence Scale Fourth Edition (WAIS-IV-NL) Technische handleiding. 2013.

15. Bouma A, Mulder J, Lindeboom J SB. Handboek neuropsychologische diagnostiek. Amsterdam: Pearson; 2012.

16. Drane DL, Yuspeh RL, Huthwaite JS, Klingler LK. Demographic characteristics and normative observations for derived-Trail Making Test indices. Neuropsychiatry Neuropsychol Behav Neurol. 2002;15(1):39–43.

17. D.C. Delis EK, Kramer. JH. D-KEFS Color Word Interference Test, Nederlandse bewerking door I. Noens en van der I. Berckelaer-Onnes. 2008;

18. Harrison JE, Buxton P, Husain M, Wise R. Short test of semantic and phonological fluency: Normal performance, validity and test-retest reliability. British Journal of Clinical Psychology. 2000;39(2):181–91.

19. Schmand B, Groenink SC, Van Den Dungen M. Letterfluency: Psychometric properties and Dutch normative data. Tijdschr Gerontol Geriatr. 2008;39(2):64–76.

20. Benedict RHB, Schretlen D, Groninger L, Brandt J. Hopkins Verbal Learning Test – Revised: Normative Data and Analysis of Inter-Form and Test-Retest Reliability. The Clinical Neuropsychologist. 1998;12(1):43–55.

21. Brandt J, Ralph H B Benedict. Hopkins Verbal Learning Test - Revised Professional Manual. 2001;

22. Tremblay MP, Potvin O, Callahan BL, Belleville S, Gagnon JF, Caza N, et al. Normative data for the rey-osterrieth and the taylor complex figure tests in Quebec-French people. Archives of Clinical Neuropsychology. 2015;30(1):78–87.

23. Lindeboom J, Schmand B, Meyer S, Jos de Jonghe. Visual Association Test Manual. 2012;

24. Van Der Elst W, Van Boxtel MPJ, Van Breukelen GJP, Jolles J. Normative data for the Animal, Profession and Letter M Naming verbal fluency tests for Dutch speaking participants and the effects of age, education, and sex. Journal of the International Neuropsychological Society. 2006;12(1):80–9.

25. Grooved Pegboard Test User Instructions. 2002;

26. Tamkin AS, Rebecca Jacobsen. Age-related norms for the Hooper Visual Organization Test. J Clin Psychol. 1984;40(6):1459–63.

27. Dodich A, Cerami C, Canessa N, Crespi C, Marcone A, Arpone M, et al. Emotion recognition from facial expressions: A normative study of the Ekman 60-Faces Test in the Italian population. Neurological Sciences. 2014;35(7):1015–21.

28. Paxton JL, Peavy GM, Jenkins C, Rice VA, Heindel WC, Salmon DP. Deterioration of visual-perceptual organization ability in Alzheimer’s disease. Cortex. 2007;43(7):967–75.

29. Relton C, Torgerson D, O’Cathain A, Nicholl J. Rethinking pragmatic randomised controlled trials: introducing the “cohort multiple randomised controlled trial” design. BMJ. 2010 Mar 24;340(mar19 1):c1066–c1066.

30. Benjamini Y, Hochberg Y. Controlling the False Discovery Rate: A Practical and Powerful Approach to Multiple Testing. J R Stat Soc Series B Stat Methodol. 1995 Jan 1;57(1):289–300.
